# Engineering characterisation of single-use bioreactor technology for mammalian cell culture applications

**DOI:** 10.1186/1753-6561-7-S6-P91

**Published:** 2013-12-04

**Authors:** Akinlolu Odeleye, Gary J Lye, Martina Micheletti

**Affiliations:** 1 Department of Biochemical Engineering, University College London, London, WC1E 7JE, UK

## Background

The commercial success of mammalian cell-derived recombinant proteins has fostered an increase in demand for novel single-use bioreactor (SUB) systems, that facilitate greater productivity, increased flexibility and reduced costs. Whilst maintaining auspicious mixing parameters, these systems exhibit fluid flow regimes unlike those encountered in traditional glass/stainless steel bioreactors. With such disparate mixing environments between SUBs currently on the market, the traditional scale-up procedures applied to stirred tank reactors (STRs) are not adequate. The aim of this work is to conduct a fundamental investigation into the hydrodynamics of single-use bioreactors at laboratory scale to understand its impact upon the growth, metabolic activity and protein productivity of an antibody-producing mammalian cell culture.

## Materials and methods

This work presents a study characterising the macro-mixing, fluid flow pattern, turbulent kinetic energy (TKE), energy dissipation rates (EDRs), and shear stresses within these bioreactor systems carried out using 2-dimensional Particle Image Velocimetry (PIV). PIV enables acquisition of whole-field flow characteristics through instantaneous velocity measurements. The SUBs employed in the PIV measurements include the 3L CellReady (Merck Millipore), PBS Biotech's PBS 3 bioreactor and the Sartorius 2L BIOSTAT Cultibag RM.

The CellReady is a stirred tank bioreactor (3 litre volume), housing a 3-bladed upward-pumping marine scoping impeller. The PIV study was conducted using the actual vessel which has an internal diameter (D_T_) of 137mm and height (H_T_) of 249mm. The marine scoping impeller (D_I_) is 76.2mm in diameter and is located near the bottom with a clearance of 30mm from the base. Measurements were obtained at varying impeller rates from 80 to 350rpm (corresponding to *Re *= 8699 to 38057). The PBS 3 is a pneumatically driven bioreactor (3 litre volume) whose mixing is induced through the buoyancy of bubbles. PIV measurements were again obtained utilising the actual PBS 3 vessel in the central vertical plane of the bioreactor at wheel speeds of 20, 27, 33 and 38rpm. The Sartorius Cultibag RM is a rocked bag bioreactor with a 2 litre total volume. A custom-made Sartorius Cultibag mimic and rocking platform was manufactured to enable the required optical access for PIV investigations. Measurements were taken at a rocking speed of 25rpm, in the vertical plane 8.5cm from the outer edge of the bioreactor. Fluid working volume (wv) was varied at 30, 40, 50 and 60% wv.

A biological study into the impact of these fluid dynamic characteristics on mammalian cell culture performance and behaviour is presented. CellReady and Cultibag cell cultures were conducted using the GS-CHO cell-line (Lonza) producing an IgG_4 _(B72.3) antibody. The impeller speed and working volume are used to vary the hydrodynamic environment within the CellReady, whilst the rocker speed is the varied parameter in the Cultibag RM.

## Results and discussion

**Figure 1:**
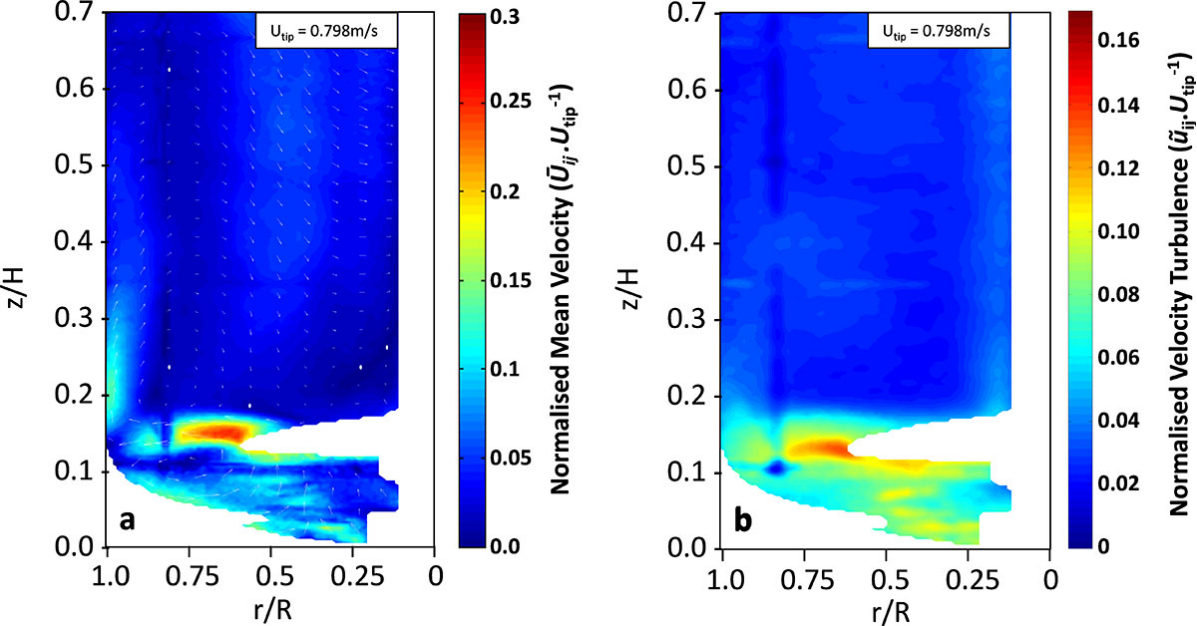
**a)** Time-resolved mean normalized velocity contour plot obtained at N = 200rpm, Re = 21747, V_L _= 2.4 L. **b) **Time-resolved turbulent velocity (${ũ}_{ij}$) contour plot obtained at N = 200rpm, Re = 21747, V_L _= 2.4 L. Resolution of 0.815mm.

The upward-pumping 3-bladed impeller within the CellReady engenders compartmentalisation of the fluid flow. This in turn contributes to the wide range of turbulence levels conveyed between the lower quarter and upper three quarters of the fluid. The maximum fluid velocity of 0.25U_tip _is achieved in the impeller discharge stream (at approximately r/R = 0.65 and z/H = 0.15) as shown in Figure 1, whilst the peak axial and radial turbulent velocities (ũ) are 0.15U_tip _and 0.11U_tip _respectively.

Disparity in cellular growth and viability throughout a range of CellReady operating conditions (80rpm-2.4L, 200rpm-2.4L and 350rpm-1L) was not substantial, although a significant reduction in cell specific productivity was found at 350rpm and 1L working volume. This is considered to be the most stressful hydrodynamic environment tested. Cells grown at these conditions displayed a metabolic shift from lactate production to net lactate consumption, without a reduction in glucose uptake. A possible reason for these observations is increased oxidative stress resulting from the higher agitation rate and gas entrainment [1,2].

The PBS exhibits a greater degree of fluid dynamic homogeneity when compared to the CellReady. Although, TKE is more than 10 times lower than values observed in the CellReady's impeller zone (which ranges from 0.0026 to 0.0455m^2^/s^2 ^at the varying impeller rates tested). Whilst TKE in the PBS peaks at approximately 0.0022m^2^/m^2 ^with a wheel speed of 38rpm, the fluid attains velocities of up to 50% of the PBS wheel speed. This corresponds to velocities of up to 15cm/s, which is within a similar range to the values observed in the CellReady.

The Sartorius RM induces fluid velocities of up to 37cm/s at 25rpm, although fluid velocity and turbulence is dominated by the radial component. EDR and TKE remain relatively low at 25rpm, with mean whole-field ensemble-averaged values of up to 0.0044m^2^/s^3 ^and 0.0020m^2^/s^2 ^respectively. These measurements are significantly lower than the mean EDR values of 0.0052 to 0.14m^2^/s^3 ^(over the RPM range of N = 80 to 350rpm) determined in the upper three quarters of the CellReady alone. Cellular response to an increase in turbulence within the rocked bag bioreactor (25 to 42rpm), results in an increase in stationary phase viable cell concentration (VCC) of 20%. In addition, cell metabolic activity and cell specific protein productivity remains relatively unchanged. The augmented homogeneity and consistency in reference to turbulence and shear stresses within the Sartorius RM may enable the cells to adapt to the more rigorous mixing, thus maintaining cell specific productivity as well as enhancing VCC. Also, cells grown in the Sartorius RM exhibit more than 60% greater cell specific productivity levels and up to 37% greater IgG_4 _titres compared to those grown in the CellReady. Even though IgG_4 _productivity increases within the Cultibag, investigations into product quality are necessary.

Given the shifts seen in metabolic behaviour and cell specific productivity, it can be concluded that the fluid dynamic environment will impact upon cellular performance. Clearly, the range of EDRs and TKEs experienced by the culture is just as pertinent as the peak turbulence levels. Therefore, determining the critical hydrodynamic parameters applicable to the different flow regimes found in SUBs, will enable greater cross-compatibility and scalability across the range of SUBs.
